# Sample, Fuzzy and Distribution Entropies of Heart Rate Variability: What Do They Tell Us on Cardiovascular Complexity?

**DOI:** 10.3390/e25020281

**Published:** 2023-02-02

**Authors:** Paolo Castiglioni, Giampiero Merati, Gianfranco Parati, Andrea Faini

**Affiliations:** 1 Department of Biotechnology and Life Sciences (DBSV), University of Insubria, 21100 Varese, Italy; 2 Laboratory of Movement Analysis and Bioengineering of Rehabilitation (Lamobir), IRCCS Fondazione Don Carlo Gnocchi ONLUS, 20148 Milan, Italy; 3 Department of Medicine and Surgery, University of Milano-Bicocca, 20126 Milan, Italy; 4 Department of Cardiovascular, Neural and Metabolic Sciences, Istituto Auxologico Italiano, IRCCS, 20145 Milan, Italy; 5 Department of Electronics, Information and Bioengineering (DEIB), Politecnico di Milano, 20131 Milan, Italy

**Keywords:** multiscale entropy, spinal cord injury, posture, autonomic nervous system, SampEn, FuzzyEn, DistEn

## Abstract

Distribution Entropy (DistEn) has been introduced as an alternative to Sample Entropy (SampEn) to assess the heart rate variability (HRV) on much shorter series without the arbitrary definition of distance thresholds. However, DistEn, considered a measure of cardiovascular complexity, differs substantially from SampEn or Fuzzy Entropy (FuzzyEn), both measures of HRV randomness. This work aims to compare DistEn, SampEn, and FuzzyEn analyzing postural changes (expected to modify the HRV randomness through a sympatho/vagal shift without affecting the cardiovascular complexity) and low-level spinal cord injuries (SCI, whose impaired integrative regulation may alter the system complexity without affecting the HRV spectrum). We recorded RR intervals in able-bodied (AB) and SCI participants in supine and sitting postures, evaluating DistEn, SampEn, and FuzzyEn over 512 beats. The significance of “case” (AB vs. SCI) and “posture” (supine vs. sitting) was assessed by longitudinal analysis. Multiscale DistEn (mDE), SampEn (mSE), and FuzzyEn (mFE) compared postures and cases at each scale between 2 and 20 beats. Unlike SampEn and FuzzyEn, DistEn is affected by the spinal lesion but not by the postural sympatho/vagal shift. The multiscale approach shows differences between AB and SCI sitting participants at the largest mFE scales and between postures in AB participants at the shortest mSE scales. Thus, our results support the hypothesis that DistEn measures cardiovascular complexity while SampEn/FuzzyEn measure HRV randomness, highlighting that together these methods integrate the information each of them provides.

## 1. Introduction

In the last decades, the interest in the entropy of heart rate time series has risen steadily due to the possibility of obtaining information on complexity aspects of cardiovascular dynamics and their alterations with disease [1]. This interest was ignited by the work of S.M. Pincus [2,3] who in 1991 proposed a computationally practical way to estimate the Kolmogorov–Sinai (K-S) entropy, which is the rate of information produced by dynamical systems. This method, called approximate entropy (ApEn), was aimed at overcoming the limits related to the computational demands and strong dependence on noise of the Grassberger–Procaccia [4] and Takens [5] formula by approximating their calculus (hence the ApEn name). The method considered segments of *m* samples as the coordinates of points in an *m*-dimensional space and evaluated how many segments were similar to each other, which means that they appeared as points closer than a given distance *r*. Then, the method evaluated how many similar segments remained similar when the dimension increased to *m* + 1. The number of similar segments can only decrease (or remain the same) from *m* to *m* + 1 and ApEn was calculated from the rate of this decrease. In this way, ApEn measured how unpredictable the value of a new sample is, given the *m* values of its preceding samples. This approach made possible the practical estimation of entropy from a relatively short series (hundreds of beats). However, ApEn was a biased estimator. In 2000, J.S. Richman and J.R. Moorman corrected this problem by introducing the Sample Entropy (SampEn) method which excludes self-matches from the count of similar segments [6]. ApEn and SampEn provide similar estimates for relatively long series while for short series ApEn gives lower entropy values than SampEn. 

Later, W. Chen et al. abandoned the dichotomous classification of “similar” or “dissimilar” segments introducing less rigid criteria based on fuzzy functions, mitigating the arbitrariness of the threshold choice, and making the estimates statistically more stable. They called their method Fuzzy entropy (FuzzyEn) [7,8]. SampEn and FuzzyEn, like ApEn from which they are derived, estimate the K-S entropy of physiological systems by evaluating a conditional probability and by approximating the original formulas to analyze short series.

More recently, P. Li et al. proposed a different approach called Distribution Entropy (DistEn) [9]. Their motivation was to remove the arbitrary choice of the distance threshold and to improve further the statistical consistency for analyzing shorter series. Like SampEn and FuzzyEn, DistEn considers segments of *m* samples as points in the *m*-dimensional space and evaluates the distances between points. However, differently from SampEn and FuzzyEn, neither a threshold nor an additional space dimension is considered because DistEn is obtained from the relative frequencies of the probability distribution function of the distances. 

This procedure makes DistEn intrinsically different from SampEn or FuzzyEn. While SampEn and FuzzyEn estimate the entropy of the time series, DistEn estimates the entropy of a structure of the phase space of the system, i.e., the distances between points of the phase space. This suggested that DistEn could be a better estimator of the “complexity structure” of the cardiovascular system than SampEn or FuzzyEn, by contrast, with more focus on the unpredictability of the series [9,10]. In this regard, the difference between “time-series unpredictability” and “system complexity”, although not rigorously defined in mathematical terms, was clearly enunciated by Costa et al. [11]. This was done to describe the apparent inconsistency of certain diseases, such as atrial fibrillation, that have similar or even higher heart-rate entropy than healthy systems, while it is expected that these disease conditions should be associated with a lower complexity of the cardiovascular system. The inconsistency could be explained by the higher randomness of heart rate during atrial fibrillation, similar to the increase of entropy in surrogate data when time series generated by complex dynamical systems are randomized. To solve the inconsistency, these authors proposed a multiscale evaluation of entropy which takes into account the entropic structure of the temporal fluctuations [11]. Successively, DistEn was proposed as an alternative approach to distinguish between time-series randomness and system complexity based on the spatial structure of the vector distances rather than on the temporal structure investigated by multiscale entropy [9].

In a previous study, DistEn performed better than SampEn in distinguishing the expected alterations of cardiovascular complexity associated with aging or heart rhythm disturbances from short data segments [12], a result that may depend on more efficient estimates because DistEn exploits all the data (not only those with distance closer than the selected threshold) and is less sensitive than SampEn to the choice of the analysis parameters. However, this result might also indicate that DistEn is better focused than SampEn on the features of the complex cardiac dynamics altered by aging or cardiac diseases. 

Our study aims to explore the nature of the information on heart rate variability (HRV) provided by DistEn and the differences with SampEn or FuzzyEn, over multiple scales. In perspective, this may allow the early diagnosis of alterations of circulatory regulation due to disease and the monitoring of the dynamics of the cardiovascular system during treatments or rehabilitation programs. For this aim, we consider two experimental conditions, one in which we expect changes in the randomness of HRV not associated with structural alterations in the generating cardiovascular system; the other, in which we expect differences in the HRV complexity associated with a structurally impaired integrative autonomic control of the circulation. By autonomic integrative control, we mean the role played by the autonomic nervous system to coordinate the heart action and the vasomotor changes in response to the ever-changing organs and body needs, and the way it interfaces with the centers of the brain, medulla, and spinal cord to organize and control body reactions and behavior [13]. Therefore, we consider the dataset of heart rate recordings in supine and sitting postures that we collected in able-bodied (AB) and spinal cord injured (SCI) individuals with a low lesion level previously [14]. To understand the rationale for the choice of this dataset, we should consider that the spinal cord injury represents a clear alteration of the overall cardiovascular control. By interrupting the flow of information between vascular districts below the lesion level and the higher brain centers, the mechanisms of integrative control are weakened allowing local regulations of individual vascular districts to prevail. Actually, we previously demonstrated that the complexity structure of HRV, as quantified by multifractality, is altered in SCI individuals [14]. By contrast, the postural shift from supine to sitting does not induce any structural alteration in the cardiovascular control and its influence on the HRV dynamics is the consequence of a relatively modest modulation of the cardiac autonomic control, consisting of increased sympathetic tone and decreased vagal tone. Selective pharmacological autonomic blockade in humans demonstrated that the short-term self-similar structure of vagal driven fluctuations of heart rate resembles the unpredictable white noise dynamic while sympathetic driven fluctuations of heart rate show much more predictable, Brown-noise like, long-term correlations [15]. Thus, the level of unpredictability of HRV in our participants should reflect the mixture of vagal and sympathetic cardiac outflows. Consequently, the HRV randomness is expected to decrease from supine to sitting, in parallel with the decrease of vagal tone and the increase in sympathetic tone, with negligible effects on the system complexity.

Therefore, this dataset allows testing two working hypotheses separately, possibly understanding the nature of the information provided by these entropy estimators better. First, assuming that a postural shift from supine to sitting does not alter the complexity structure of the cardiovascular control but only the irregularity/unpredictability of the heart-rate series by modulating the autonomic tone, we will test the hypothesis that postural differences will be clearer for SampEn or FuzzyEn than for DistEn (preliminary results on this issue have been presented at the 12th ESGCO Conference in 2022 [16]). Second, assuming that in SCI individuals, previously associated with an altered multifractality, the impaired integrative autonomic regulation influences the cardiovascular complexity, we will test the hypothesis that DistEn reveals more pronounced and significant differences between AB and SCI groups than SampEn or FuzzyEn.

## 2. Materials and Methods

### 2.1. Synthetized Series

To compare the general characteristics of SampEn, FuzzyEn, and DistEn, we generated 30 series for each of 3 stochastic processes: Gaussian white, pink, and brown noises. Furthermore, by using the logistic recursion *x*(*n* + 1) = ω × *x*(*n*) × (1 − *x*(*n*)) we generated 30 chaotic (ω = 4) and 30 periodic (ω = 3.5) series. Each of the 5 × 30 synthesized series was composed of 512 samples. 

### 2.2. Subjects and Data Collection

We considered the 14 SCI individuals with a complete lesion (ASIA scale A) at a level between the 12th thoracic and 4th lumbar vertebra and 34 AB controls who participated in our previous study for evaluating spectral and fractal HRV components [17]. Paraplegic individuals had complete traumatic lesions with no current or previous history of overt dysautonomia (details on exclusion criteria are reported in [18]). Due to the low level of the lesion, the paraplegic participants had impaired autonomic control of organs innervated by the pelvic nerve and by efferent pathways from the mesenteric ganglia but intact autonomic cardiac control. The experimental protocol, which is described in detail in [18], consisted in recording the ECG at 200 Hz sampling rate for 10 min in sitting and 10 min in supine postures after an adaptation period of 5 min, in a quiet environment (for technical reasons, the supine recording was missing in two SCI participants). The study was approved by the ethics committee of IRCCS Don C. Gnocchi Foundation, Milan (Italy) and each subject gave informed consent before the start of the experiment.

AB and SCI groups were matched by age (AB, 39.3 ± 12.1 years; SCI, 40.9 ± 10.0 years; mean ± SD), body mass index (AB, 24.7 ± 2.6; SCI, 25.5 ± 4.8 kg·m^−2^) and female/male ratio (AB, 5/29; SCI: 2/12). As demonstrated previously [17] (p. 6), the mean R-R interval (RRI) did not differ significantly between groups (supine AB = 958 ± 26 ms, SCI = 885 ± 40 ms; sitting AB = 864 ± 23 ms, SCI = 853 ± 34 ms, median ± SE median); furthermore, AB and SCI participants were also matched in terms of the time-domain vagal indexes RMSSD (supine AB = 30 ± 4 ms^2^, SCI = 28 ± 6 ms^2^; sitting AB = 27 ± 3 ms^2^; SCI = 28 ± 5 ms^2^) and pNN50 (supine AB = 7.8 ± 3.6%, SCI = 8.8 ± 3.3%; sitting AB = 6.2 ± 2.1%, SCI = 9.9 ± 3.7%), as well as frequency-domain vagal and sympatho/vagal indexes, HF power (supine AB = 258 ± 90 ms^2^, SCI = 132 ± 84 ms^2^; sitting AB = 194 ± 47 ms^2^, SCI = 179 ± 80) and LF/HF powers ratio (supine AB = 2.3 ± 0.3, SCI = 2.9 ± 1.2; sitting AB = 3.9 ± 0.4, SCI = 3.5 ± 0.7). 

### 2.3. Entropy Estimators

Premature beats visually identified on the RRI time series from the ECG were removed. The HRV entropy was estimated over a segment of 512 consecutive beats, with embedding dimensions *m* = 1 and *m* = 2.

As to SampEn and FuzzyEn, we set the threshold *r* equal to 20% of the standard deviation of the series. SampEn was calculated as in [6]: given the *N* samples **X** = {*x*_1_ *x*_2_ *… x_N_*} we constructed the template vectors for the dimension *m*,

(1) $$X_{i}^{m}={\left[x_{i},x_{i+1},\ldots ,x_{i+m-1}\right]}^{T}\;1\leq i\leq N-m,$$

calculated the infinity norm distance between vectors

(2) $$d_{ij}^{m}=\|X_{i}^{m}-X_{j}^{m}\|{}_{\infty }\;1\leq i,j\leq N-m,\;j\geq i+1,$$

and counted the pairs of vectors with a distance lower than *r*, *n_p_*(*m,r*). We repeated the same steps for *m* + 1 obtaining:

(3) $$SampEn\left(X,N,m,r\right)=-ln\frac{n_{p}\left(m+1,r\right)\;}{n_{p}\left(m,r\right)}$$

FuzzyEn was calculated as in [8]: first, we obtained the average of the similarity degree among vectors $X_{i}^{m}$ as

(4) ϕm=∑i=1N−m(1N−m−1∑j=1,j≠iN−mexp(−dijmnr))

with *n* = 2. In Equation (4) we employed an exponential membership function following [19] but different choices might imply different behaviors of the estimator [8]. Then we calculated the same quantity for *m* + 1 and Fuzzy Entropy as:

(5) $$FuzzyEn\left(X,N,m,r,n\right)=ln\left[\phi^{m}\left(n,r\right)\right]-ln\left[\phi^{m+1}\left(n,r\right)\right]$$

It should be noted that the original definition of FuzzyEn [7] removes local trends making FuzzyEn not directly comparable to SampEn at the same *m* [20]. For this reason, we estimated the global FuzzyEn without trend removals as introduced in [8]. 

DistEn was obtained as in [9] by calculating the empirical probability distribution function (ePDF) of the $d_{ij}^{m}\;$ distances. We calculated ePDF on *M* = 512 equispaced bins over the range of the distance values, its Shannon entropy ShEn as

(6) $$ShEn=-\overset{M}{\underset{t=1}{\sum }}p_{t}log_{2}\left(p_{t}\right)$$

and

(7) $$DistEn\left(m\right)=\frac{ShEn\left(m,M\right)}{log_{2}\left(M\right)}$$

**Figure 1:**
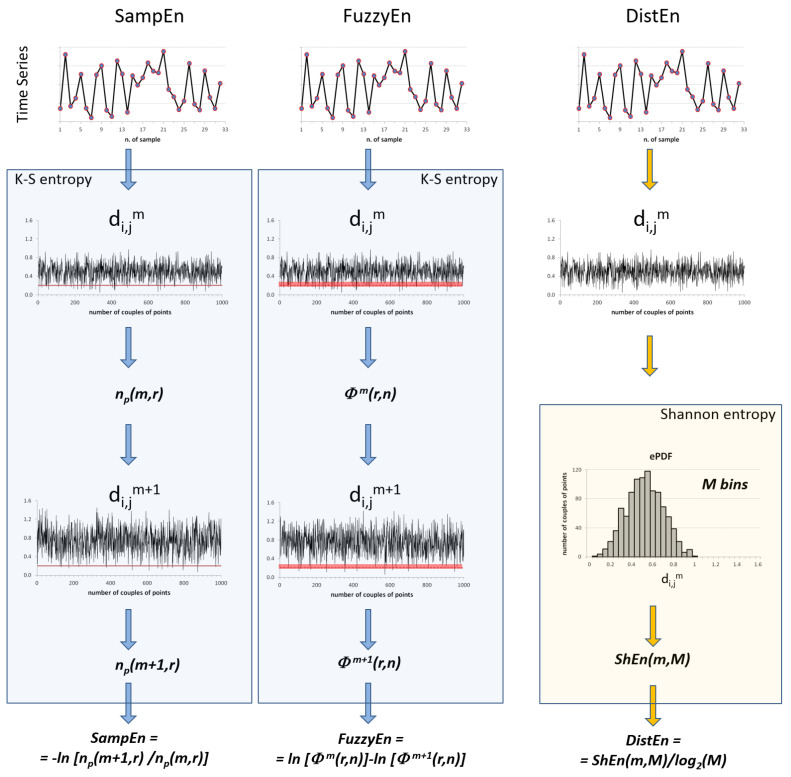
Scheme of Sample (SampEn, **left**), Fuzzy (FuzzyEn, **center**), and Distribution Entropy (DistEn, **right**) calculation. SampEn and FuzzyEn estimate the K-S entropy of the series by representing segments of *m* and *m +* 1 samples as points in spaces of *m* and *m* + 1 dimensions and calculating the distances between points in both spaces, d_i,j_*^m^* and d_i,j_*^m+^*^1^, based on the conditional probability that similar segments of *m* samples remain similar when the segment length increases to *m +* 1. SampEn is the negative logarithm of the ratio between the number of points *n_p_* closer than the threshold *r* (red line) in spaces of *m +* 1 and *m* dimensions. FuzzyEn is the difference between the logarithms of the degree of similarity among points in the *m* and *m* + 1 spaces, *Φ^m^*(*r,n*) and *Φ^m+^*^1^(*r,n*), with *r* the fuzzy distance (red bar) and *n* the exponent that defines the similarity function. Unlike SampEn and FuzzyEn, DistEn does not calculate the entropy of the series but the Shannon entropy (ShEn) of the distances, d_i,j_*^m^*, by estimating the empirical probability distribution function (ePDF) of d_i,j_*^m^* over *M* bins and ShEn as $-\overset{M}{\underset{t=1}{\sum }}p_{t}log_{2}\left(p_{t}\right)$, with *p_t_* the relative frequency of bin *t*.

Figure 1 schematizes the steps for the calculus of these three entropy methods.

Multiscale estimates of SampEn (mSE), FuzzyEn (mFE), and DistEn (mDE) at scales τ between 2 and 20 beats were obtained after low-pass filtering the RRI series at each τ by a zero-phase Butterworth filter with a cut-off frequency equal to 0.5/τ as in [21]. We used this filter because of its better transition band compared to the moving average originally proposed for coarse-graining [22]. Then the coarse-graining decimation (consisting in taking one sample for every τ samples) was not applied to use all the samples of the low-pass filtered series to improve the quality of the estimate [21], and we calculated mSE, mFE, and mDE setting a delay equal to τ between consecutive samples of the template vectors as defined in [23]. 

### 2.4. Statistics

As stated in the introduction, the aim is to evaluate whether Sample, Fuzzy and Distribution entropy identify or not the expected HRV changes due to (1) the postural autonomic activation; and (2) the impaired integrative autonomic control. This logically leads to testing two null hypotheses for each entropy estimator, one regarding the significance of the factor “posture” comparing supine and sitting positions, the other regarding the significance of the factor “case” comparing AB and SCI participants.

To test these hypotheses for SampEn, FuzzyEn, and DistEn, we applied a Linear Mixed-Effects Model that simultaneously provides the statistical significance of the factors Case (AB vs. SCI) and Posture (Supine vs. Sitting) and their interaction. We tested if the residuals were normally and equally distributed and rank-transformed the data if the assumptions were not satisfied. When one of the factors or their interaction was significant at *p* < 0.05, we tested the differences between Supine and Sitting for each group and the differences between AB and SCI participants in each posture with a-posteriori contrasts, accounting for multiple comparisons with the false discovery rate correction. 

As to the multiscale entropies, the statistical tests should regard each of the 19 scales from τ = 2 to τ = 20 separately. For conciseness, we employed a non-parametric approach (Wilcoxon–Mann–Whitney test) to check the assumption of Linear Mixed-Effects Models for each scale. A null hypothesis was tested separately at each scale and thus corrections for multiple comparisons were not applied. The ability of mSE, mFE, and mDE to detect HRV changes associated with posture was assessed by the Wilcoxon Signed Rank test comparing Supine and Sitting positions by groups at each τ; the ability to detect HRV changes associated with the impaired integrative autonomic regulation was assessed by the Wilcoxon Rank Sum test comparing AB and SCI groups by posture at each τ.

## 3. Results

### 3.1. Synthetized Series

**Figure 2:**
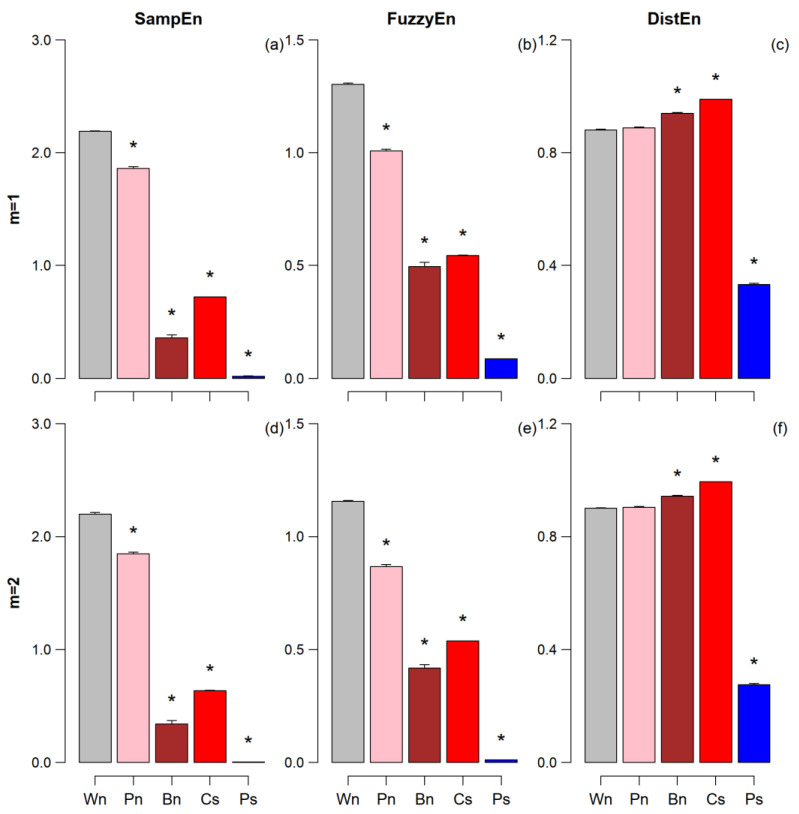
Mean and SEM of SampEn (panels (**a**,**d**)), FuzzyEn (panels (**b**,**e**)), and DistEn (panels (**c**,**f**)) over groups of 30 synthesized series for dimensions *m* = 1 and *m* = 2. Wn = white noise; Pn = pink noise; Bn = Brown noise; Cs = chaotic series; Ps = periodic series. The * indicates differences vs. Wn at *p* < 0.05.

Figure 2 compares the entropy estimates for the synthesized series. Synthetized series allow a better understanding of the role of SampEn and FuzzyEn as estimators of time series irregularity and DistEn as an estimator of system complexity. In fact, the figure highlights a very different behavior of the two estimators of the K-S entropy, SampEn, and FuzzyEn, compared to DistEn. SampEn and FuzzyEn provide the largest entropy for white noise (the more unpredictable series) and lower values for pink and brown noises, reflecting the increase in the long-range correlation of these series. The entropy of the chaotic series is in between pink- and brown-noise entropies, and the periodic series has the lowest entropy, close to zero. 

By contrast, DistEn provides a different picture and distribution entropy is the largest for the chaotic series. Furthermore, DistEn is greater (and not lower) for Brown than white noise. Moreover, pink and white noises virtually have the same DistEn. The periodic series has the lowest DistEn, as for SampEn/FuzzyEn. However, while SampEn/FuzzyEn of the periodic series is just 1% of the value of white noise, DistEn of the periodic series is around one-third of the white-noise DistEn.

**Figure 3:**
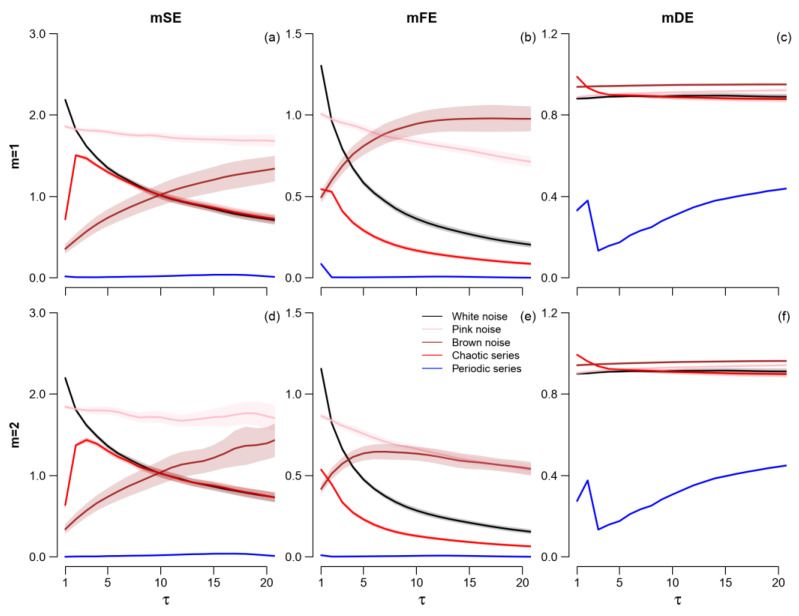
Multiscale Sample (mSE, panels (**a**,**d**)), Fuzzy (mFE, panels (**b**,**e**)), and Distribution (mDE, panels (**c**,**f**)) Entropies of synthesized signals. Values as mean and 95% confidence intervals.

As to the multiscale entropy (Figure 3), mSE and mFE show almost the same trends: white and pink noises and the chaotic series monotonically decrease with the scale for all τ > 2, with a steeper decrease for mFE; the periodic series is close to zero; and the Brown noise entropy increases with τ, although it happens at all the scales for mSE and up to τ = 10 only for mFE. The estimates appear slightly more stable for mFE than mSE, and for *m* = 1 than *m* = 2. However, there are differences between mSE and mFE in the relative entropy values among signals. For instance, mSE is greater for pink than Brown noise at all the scales while for mFE the difference disappears at τ > 10 for *m* = 2 and is even reversed (with mFE greater for Brown than pink noise) for *m* = 1. Furthermore, white noise and the chaotic series have the same mSE at τ > 5 for mSE, while mFE is consistently greater for white noise than the chaotic series at all τ. 

The profile of mDE as a function of the scale appears rather different. The mDE of the chaotic series decreases very quickly with τ so that Brown and pink noises have a greater mDE than the chaotic series at the larger scales and Brown noise has the largest mDE at all τ > 2. The periodic series has the lowest mDE at all scales. It should be noted that the mDE of the periodic series generated by the logistic recursion with ω = 3.5 was calculated in two previous works [24,25] that reported a systematic periodicity at scale multiples of τ = 4 which, however, is absent in our estimates of Figure 3c,f. The reason is likely due to the poor transition band of the moving average filter employed in the previous works before coarse-graining as demonstrated in Appendix A.

### 3.2. Real Beat-to-Beat Series

#### 3.2.1. SampEn, FuzzyEn, and DistEn

**Figure 4:**
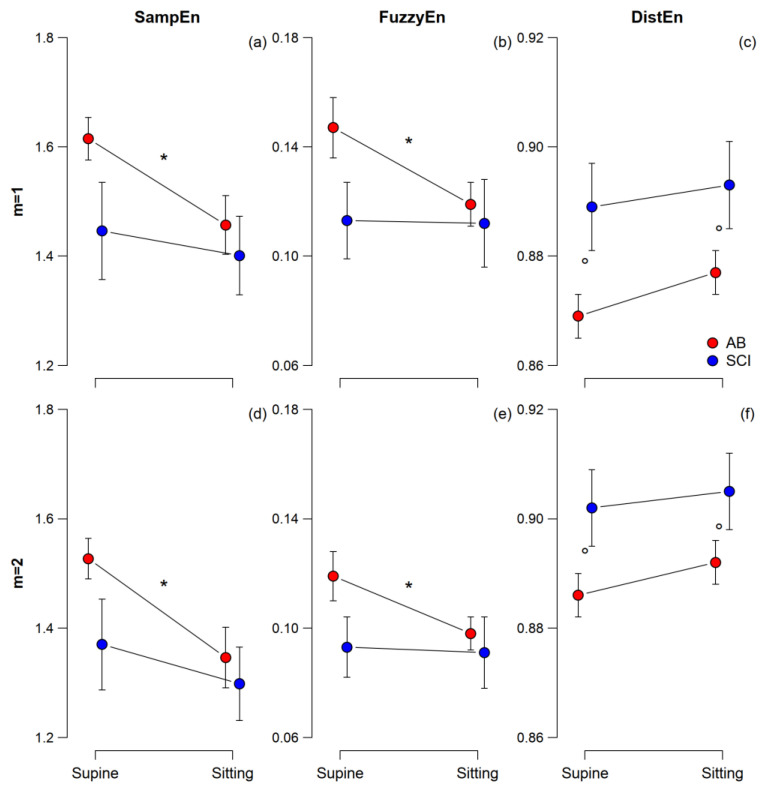
Mean and SEM of SampEn (panels (**a**,**d**)), FuzzyEn (panels (**b**,**e**)), and DistEn (panels (**c**,**f**)) in AB and SCI participants for *m* = 1 and 2. The * indicates significant differences between postures or groups at *p* ≤ 0.05; the ° indicates statistical trends at *p* < 0.10.

Table 1 reports the significance of “Posture” and “Case” factors and their interaction. Only “Posture” is significant for SampEn and FuzzyEn. In particular, Figure 4 shows lower SampEn and FuzzyEn in Sitting. The percent decrease of entropy from Supine to Sitting is more pronounced for the AB group both when quantified by SampEn (*m* = 1: AB = −9.9%, SCI = −6.2%; *m* = 2: AB = −11.8%, SCI = −5.1%) and FuzzyEn (*m* = 1: AB = −19%, SCI = −0.9%; *m* = 2: AB = −17.6%, SCI = −2.2%). The differences reach statistical significance in the AB group only. 

By contrast, Table 1 reports that neither “Posture” nor the interaction of “Posture” with “Case” is a significant factor for DistEn. However, differently from SampEn or FuzzyEn, the “Case” factor is significant for DistEn demonstrating that the low-level spinal cord lesion influences cardiovascular complexity. In particular, Figure 4 provides evidence of higher DistEn in SCI than AB participants. The difference between SCI and AB groups expressed as a percentage of the AB value was similar in the two postures (*m* = 1: Supine = +2.3%, Sitting = +1.8%; *m* = 2: Supine = +1.8%, Sitting = +1.5%). 

#### 3.2.2. mSE, mFE and mDE

**Figure 5:**
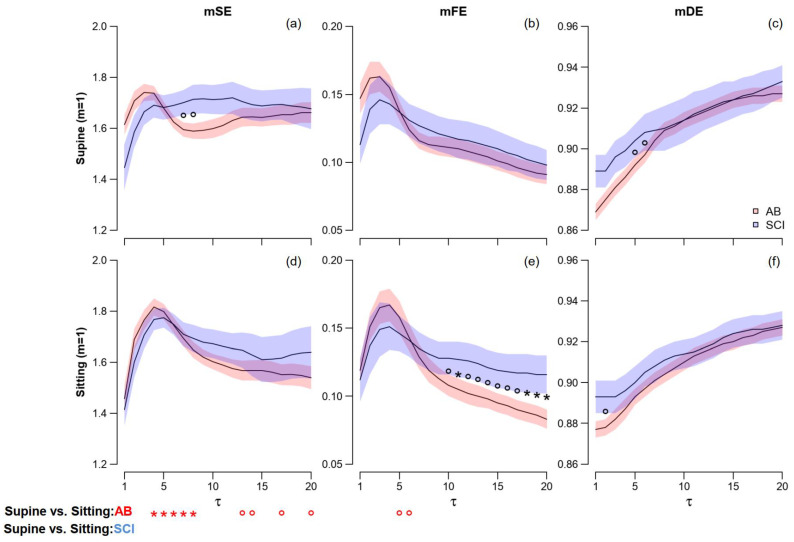
Multiscale Sample (panels **a**,**d**), Fuzzy (**b**,**e**), and Distribution Entropies (**c**,**f**) for *m* = 1 in supine and sitting postures. Values as mean ± SEM over SCI and AB controls. The symbols refer to the scale-by-scale statistics for 2 ≤ τ ≤ 20: the * and ° in the panels a–f indicate significant differences (*p* ≤ 0.05) or trends (*p* < 0.10) between AB and SCI groups; and in the panels (**d**–**f**) indicate significant differences (*p* ≤ 0.05) or trends (*p* < 0.10) between postures.

**Figure 6:**
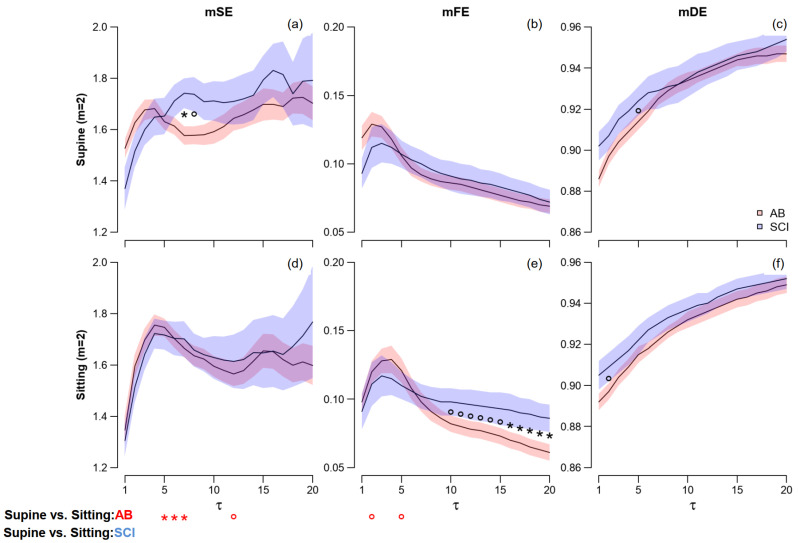
Multiscale Sample (panels **a**,**d**), Fuzzy (**b**,**e**), and Distribution Entropies (**c**,**f**) for *m* = 2 in supine and sitting postures. Values as mean ± SEM over SCI and AB controls. The symbols refer to the scale-by-scale statistics for 2 ≤ τ ≤ 20: the * and ° in the panels a–f indicate significant differences (*p* ≤ 0.05) or trends (*p* < 0.10) between AB and SCI groups; and in the panels (**d**–**f**) indicate significant differences (*p* ≤ 0.05) or trends (*p* < 0.10) between postures.

Figure 5 and Figure 6 show the profiles of multiscale entropies separately in the two groups and postures, respectively, for embedding dimensions 1 and 2. The capability of DistEn to discriminate between AB and SCI groups is almost lost at τ > 1 and mDE shows just a weak tendency to separate the AB and SCI groups at τ < 8. At greater mDE scales, the two groups overlap each other. 

By contrast, the multiscale Fuzzy Entropy allows distinguishing between the AB and SCI groups at scales τ > 10 but only in the sitting position. Differences between groups that are significant (*m* = 2) or close to the significance level (*m* = 1) appear for mSE too around τ = 7: in this case, however, the trends characterize the supine position only.

Significant differences between postures are detected only by mSE: they regard the AB group only and scale τ ≤ 8.

## 4. Discussion

Distribution entropy has been originally proposed to describe complexity aspects of the physiological systems generating HRV because Sample entropy was considered a measure more focused on the heart-rate irregularity [9,10]. Our study contributes to clarifying this aspect. It is designed to compare how Distribution, Sample and Fuzzy entropies quantify HRV changes having different origins: one is a posture change that modifies HRV without affecting the physiological structure underlying the cardiovascular complexity; the other is a condition anatomically characterized by an impaired integrative regulation. 

This comparison was possible considering a human model of impaired integrative autonomic control: the paraplegic individual with a low-level spinal lesion. Paraplegic subjects with a high-level lesion, above the fifth thoracic vertebra, T5, have an impaired sympathetic outflow that also affects the neural pathways directly innervating the heart: this condition might influence both the long-term correlations of the heart rate (thus, the heart rate irregularity) [18] and the overall system complexity. Lesions between T5 and T11 do not directly affect the cardiac autonomic outflows but influence importantly the central autonomic regulation of several vascular districts. These innervations are responsible for vasomotor components that generate the Mayer waves in blood pressure, reflected in the low-frequency spectral power of HRV through the baroreflex [17]. Thus, also in the case of spinal lesions between T5 and T11, we may expect both an altered cardiovascular complexity due to the lesion and an altered heart-rate randomness due to the loss of predictable oscillatory components (the Mayer waves). By contrast, paraplegic individuals with lesions below T11 preserve the oscillatory components of HRV that characterize the power spectrum of AB controls [17]. Nevertheless, their spinal lesion alters the cardiovascular complexity as previously demonstrated by assessing the HRV multifractality [14]. Therefore, paraplegic individuals with spinal lesions below T11 represent a model of human cardiovascular regulation that allows testing the information provided by different entropy metrics, having an intact HRV power spectrum (i.e., second-order statistics) but an altered multifractal spectrum (higher-order statistics). In the following, we will discuss the results we obtained by assessing our dataset of heart rate recordings with Sample, Fuzzy, and Distribution entropy methods.

### 4.1. SampEn, FuzzyEn, and DistEn

The significances of the factors “Case” and “Posture” highlighted the different nature of DistEn compared to SampEn or FuzzyEn (Table 1) and support the hypothesis that DistEn may identify structural alterations in the cardiovascular system rather than measuring changes in the heart rate randomness, like SampEn or FuzzyEn. In line with the starting hypothesis that a postural shift is not expected to be associated with structural changes affecting the system complexity, DistEn fails to detect the increase in cardiac sympathetic tone and decrease in vagal tone that characterizes the shift from supine to sitting. These autonomic changes are detected by SampEn and FuzzyEn similarly, both describing a decrease in heart rate irregularity in sitting. In fact, the higher sympatho/vagal balance in the sitting position should increase the relative amplitude of the slower HRV components driven by the cardiac sympathetic outflow, increasing the long-range correlation of heart rate values and consequently their beat-to-beat predictability. Our results also show that both SampEn and FuzzyEn quantify a more marked postural decrease of entropy in the AB group. The similarity of the results provided by SampEn and FuzzyEn is not surprising because both count the number of similar segments of length *m* that remain similar when the segment length increases to *m* + 1, even if the way this number is obtained and processed differs between the methods. 

Also in line with the starting hypothesis is our finding that DistEn, unlike SampEn or FuzzyEn, distinguishes the altered integrative autonomic control in SCI participants (the “Case” factor is significant for DistEn only, Table 1). The effect is a greater DistEn in the SCI group. We cannot exclude that SampEn and FuzzyEn may also partially reflect the effect of the spinal lesion on the system complexity and Figure 4 might suggest a tendency for SampEn/FuzzyEn to separate the AB and SCI groups in Supine. However, the difference is far from being significant, supporting the hypothesis that SampEn and FuzzyEn are more sensitive to changes in the signal randomness, and DistEn to alterations in the system complexity, as formulated in [9,10].

**Figure 7:**
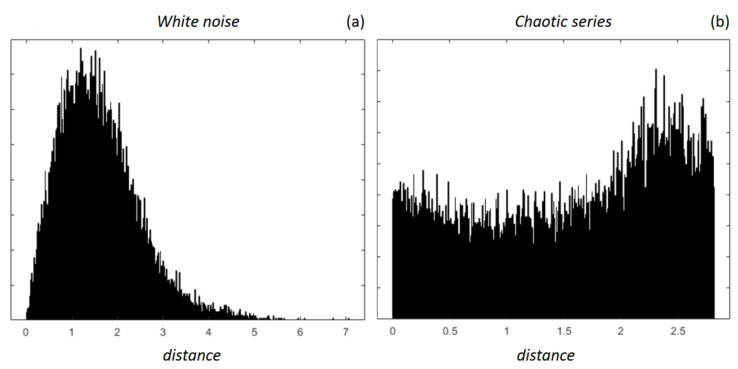
Empirical probability distribution functions of distances between vectors at *m* = 2 for white noise series (panel (**a**)) and chaotic series (panel (**b**)).

Our results, therefore, indicate that the HRV complexity, as quantified by DistEn, is greater in the SCI group. We cannot indicate how the impairment of the autonomic integrative control due to the spinal cord lesion may have produced an increased complexity. However, our results on the synthesized series might help to formulate a tentative hypothesis. The trends we observed in our AB and SCI volunteers have the same sign of the differences between white noise and chaotic series: greater DistEn and lower SampEn/FuzzyEn for the chaotic series compared to white noise, as in SCI compared to AB participants. Looking at the ePDF functions of white noise and chaotic series (e.g., see Figure 7) we found that the chaotic dynamics produced distances equally distributed at all the amplitudes; by contrast, even if white noise is totally unpredictable the distances between its vectors have certain amplitudes more likely than others. This might suggest that the presence of an effective integrative autonomic control allows the higher centers of the nervous system to coordinate the local vascular regulations orchestrating harmonically the cardiovascular dynamics and shaping the ePDF in a sort of bell curve. This would avoid that independent regulations of the local vascular districts reverberate into less structured, and flatter, ePDF, resulting in a higher DistEn.

### 4.2. mSE, mFE and mDE

The multiscale entropy approach was originally proposed to distinguish the sample entropy of the uncorrelated white noise process from the entropy of series with long-term correlation (like pink noise), as a tool for distinguishing time-series randomness from system complexity. Our results on the synthesized series confirm the ability of the multiscale approach to distinguish between white and pink noises not only for SampEn but also for FuzzyEn. By contrast, the multiscale approach appears much less efficient in separating white from pink noise when applied to DistEn. 

Furthermore, the multiscale approach suggests complementary roles of Sample, Fuzzy, and Distribution Entropy in describing the HRV dynamics. A result that deserves to be discussed is that at scales τ > 1, mSE and mFE provide substantially different information even if both estimate the K-S entropy by counting points close to each other in *m* and *m* + 1 dimensions. This contrasts strikingly with the similar behavior of SampEn and FuzzyEn in distinguishing between groups or postures (Figure 4).

In fact, while both SampEn and FuzzyEn distinguish similarly the posture change in the AB group, mSE only and not mFE differs significantly between Supine and Sitting in the AB group. A possible explanation is offered by the analysis of the synthesized series (Figure 3). The way mSE and mFE change with τ is similar for all the signals. However, the relative differences between signals are not the same for mSE and mFE. For instance, let us consider white and Brown noises: for both mSE and mFE, the white noise decreases with τ from its highest value at τ = 1 and the Brown noise increases with τ from a much lower value at τ = 1. However, the slope of Brown noise as τ increases differs between mSE and mFE and at around τ = 4 mSE is much higher for the white than Brown noise while the mFE of white and Brown noises are similar. We might hypothesize that the fuzzy and dichotomous thresholds used for classifying segments as similar or dissimilar weight differently in strongly correlated series, such as the Brown noise. The consequence is that the mSE and mFE profiles with τ may differ depending on the level of correlation among samples, justifying why the Sample and Fuzzy methods may provide different information on the HRV entropy at scales greater than one.

The same reasoning may also explain a second interesting difference between mSE and mFE, i.e., the ability of these multiscale methods to distinguish between AB and SCI groups. In fact, at τ ≥ 10 (scale likely influenced by vasomotor components such as the Mayer waves) the mFEs of AB and SCI groups in sitting position diverge while the mSEs almost overlap each other. The greater mFE in sitting SCI participants suggests a higher system complexity, in line with the result provided by DistEn. Furthermore, differences between groups seem to appear in the supine position too, in this case for mSE around τ = 7 (scales more likely influenced by respiration). Our results, therefore, suggest that the Sample and Fuzzy entropy methods provide complementary information on HRV when considered over multiple scales.

By contrast, no significant differences between groups or postures are identified by mDE at any τ > 1. In particular, the difference between groups that characterize DistEn (i.e., mDE at τ = 1) vanishes as τ increases and the mDEs of the AB and SCI groups practically overlap each other at τ > 7. This is not completely unexpected because DistEn has been proposed to detect alterations in the system complexity even at scale 1, without requiring a multiscale analysis, as for Sample and Fuzzy entropies; and because the way mSE and mFE detect the entropic temporal structure depends critically on the definition of the threshold *r* [26], a parameter that is absent in the calculation of DistEn. However, a deterioration of the Distribution Entropy method in revealing structural alterations in the system complexity at larger scales is possible: actually, the mDE inability to detect differences between white noise and the chaotic signals at τ > 2 was reported previously [25] and confirmed by our results (Figure 3). Even if mDE proved to be valid in distinguishing multiscale entropy alterations with aging and congestive heart failure [25], likely thanks to its capacity to provide statistically consistent estimates even on very short series, the low performance in distinguishing purely random from deterministic chaotic signals at τ > 1 may be responsible for the lack of significant differences between our AB and SCI groups. Interestingly, a recent study suggested that the relatively poor performance of mDE in distinguishing between chaotic and random series at larger scales is due to neglecting more complete quantifications of the distance between vectors in the embedding space [24]. These authors introduced an improved distribution entropy method taking into account also the orientation-based angular distance and the rank-based Spearman distance between vectors in the phase space, with promising results [24]. 

### 4.3. Limitations

Some limitations should be considered in interpreting our results. The first regards the relatively small number of enrolled SCI participants, in particular, female participants. A larger number might have identified as significant some reported statistical tendencies, or allowed identifying sex-related differences. However, it should be considered that the enrollment criteria in the SCI group were particularly stringent, requiring a well-delimited lesion level, the completeness of the lesion, and an active lifestyle to reduce the effect of sedentariness as a confounding factor. Furthermore, some medications could not be suspended (3 SCI participants were taking antibiotics for urinary infection, and another 3 were taking drugs against muscle spasms) and a possible influence of these drugs on the results cannot be completely excluded. 

## 5. Conclusions

We may conclude that Sample, Fuzzy, and Distribution Entropy methods are not alternatives one to the other, but they complement each other providing different information on HRV. Regarding the beat-to-beat scale, DistEn cannot be considered an index of sympathovagal balance, because, unlike SampEn and FuzzyEn, it does not distinguish between supine and sitting postures either in healthy controls or in paraplegic volunteers. However, it demonstrates the ability to distinguish alterations in the integrative autonomic control that SampEn and FuzzyEn fail to detect.

At larger scales, mSE and mFE provide different pieces of information that cannot be derived from mDE and that, taken together, allow obtaining a more complete picture of the alterations induced by the posture shift and the spinal lesion. Therefore, Sample, Fuzzy, and Distribution entropies are not alternative tools but they represent different aspects of a more complex picture.

## Figures and Tables

**Table 1:** Significance p of Posture and Case factors and their interaction after linear mixed-effects model analysis for two embedding dimensions *m*.

Factor	SampEn	FuzzyEn	DistEn
*m* = 1			
Posture	0.029 *	0.024 *	0.15
Case	0.17	0.30	0.020 *
Interaction	0.22	0.13	0.94
*m* = 2			
Posture	0.007 *	0.038 *	0.25
Case	0.20	0.28	0.026 *
Interaction	0.23	0.23	0.90

Bold and * highlight statistical significances at *p* < 0.05.

**Figure A1:**
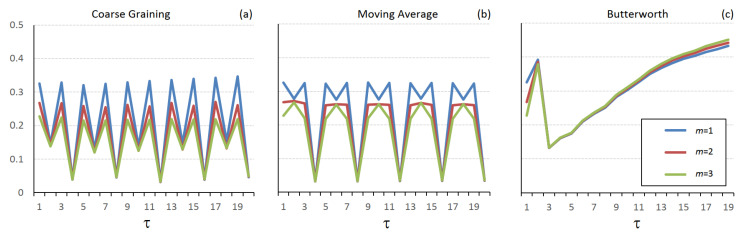
Multiscale Distribution Entropy of the periodic signal estimated with the traditional coarse-graining approach (**a**), with the delay method using the moving average filter (**b**), and the Butterworth filter (**c**).

## Data Availability

The data supporting the main findings of this study are available at URL https://doi.org/10.5281/zenodo.7491970 (accessed on 28 January 2023) with access granted on justified request to researchers meeting the criteria for access to confidential data due to the hospital research policy and restrictions requested by the ethical committee.

## Appendix A

The multiscale Distribution Entropy has been evaluated for the logistic regression with ω = 3.5 (periodic signal) and ω = 4 (chaotic signal) in two previous papers [24,25]. While mDE estimates for the chaotic signal are similar to our estimates (Figure 3c,f), the estimates of the periodic signal present a systematic oscillation repeating at scales multiple of τ = 4 that is absent in our estimates. Differently from [24,25], we used a Butterworth filter for evaluating scales τ > 1 as suggested in [22] because of its narrower transition band compared to the moving average filter used in previous estimates. To verify that the reported systematic oscillation in multiscale Distribution Entropy of the periodic signal is an artifact generated by the broad transition band of the filter, we estimated its mDE again using the classic coarse-graining approach (which consists in decimating the signal by taking one sample every τ and by substituting the selected sample with the average over τ contiguous samples) and the moving-average approach followed in [24,25] (which consists of filtering the signal with a moving average of order τ and calculating the distribution entropy by introducing the delay δ = τ among the filtered samples). Figure A1 shows that mDE estimates after coarse-graining or moving average present a clear periodicity at multiples of τ = 4 and that its values depend on the filter (e.g., for m = 3 mDE shows a relative minimum after coarse graining and a relative maximum after the moving average, at scales τ = 2 + 4*n*, with *n* = 0, 1, 2,…). This result demonstrates that the periodicity is introduced by the filtering or coarse graining method and suggests that it is an artifact introduced by the broad transition band of the moving average.
